# New Insights on Wood Dimensional Stability Influenced by Secondary Metabolites: The Case of a Fast-Growing Tropical Species *Bagassa guianensis* Aubl.

**DOI:** 10.1371/journal.pone.0150777

**Published:** 2016-03-23

**Authors:** Julie Bossu, Jacques Beauchêne, Yannick Estevez, Christophe Duplais, Bruno Clair

**Affiliations:** 1 CNRS, UMR EcoFoG, AgroParisTech, Cirad, INRA, Université des Antilles, Université de Guyane, 97310 Kourou, France; 2 Cirad, UMR EcoFoG, AgroParisTech, CNRS, INRA, Université des Antilles, Université de Guyane, 97310 Kourou, France; Monash University, AUSTRALIA

## Abstract

Challenging evaluation of tropical forest biodiversity requires the reporting of taxonomic diversity but also the systematic characterization of wood properties in order to discover new promising species for timber industry. Among wood properties, the dimensional stability is regarded as a major technological characteristic to validate whether a wood species is adapted to commercial uses. Cell structure and organization are known to influence the drying shrinkage making wood density and microfibrils angle markers of choice to predict wood dimensional stability. On the contrary the role of wood extractive content remains unclear. This work focuses on the fast-growing tropical species *Bagassa guianensis* and we report herein a correlation between heartwood drying shrinkage and extractive content. Chemical extractions and shrinkage experiments were performed on separate wood twin samples to better evaluate correctly how secondary metabolites influence the wood shrinkage behaviour. Extractive content were qualitatively and quantitatively analysed using HPLC and NMR spectroscopy. We found that *B guianensis* heartwood has a homogeneous low shrinkage along its radius that could not be explained only by its basic density. In fact the low drying shrinkage is correlated to the high extractive content and a corrected model to improve the prediction of wood dimensional stability is presented. Additionally NMR experiments conducted on sapwood and heartwood extracts demonstrate that secondary metabolites biosynthesis occurs in sapwood thus revealing *B*. *guianensis* as a *Juglans*-Type heartwood formation. This work demonstrates that *B*. *guianensis*, a fast-growing species associated with high durability and high dimensional stability, is a good candidate for lumber production and commercial purposes.

## Introduction

Tropical forests are much studied for their high plant species diversity. The creation of taxonomies, a process which has been being undertaken for centuries, still faces the challenge of this tremendous biodiversity. Unfortunately, data gathered from new species are mostly genetic, and diversity of wood properties remains poorly understood. Systematic wood characterization offers a unique opportunity to discover original materials with promising properties that could greatly impact the timber industry.

Dimensional stability is a sought after characteristic largely used as the criterion to determine wood uses. Commercial species possess tangential and radial shrinkage from 5% to 10% and 2% to 6% respectively [1]. The shrinkage variability between wood species originates from the structure and organization of cells as well as their chemical properties. At the macroscopic scale, one can observe variations in the grain angle, *ie* the wood fibres are not parallel to the trunk axis but form a helix around the tree, in some species the helix can alternate from S to Z and is named interlocked grain. At the microscopic scale variations in wood cell wall thickness directly impact the macroscopic wood basic density. Finally, cell wall composition (cellulose, lignin, extractives) and organization (angle of cellulose microfibrils helix around the cell) affect the physical and mechanical behaviour of wood. These characteristics vary among species and are known to also change during tree life stages or in different environments.

Shrinkage mechanisms were initially investigated by Newlin and Wilson [2] and volumetric shrinkage has been shown to be positively correlated to basic density [3, 4]. However the simple linear regression was later contested [5]. So far several intrinsic parameters have been shown to influence drying shrinkage.

Transverse shrinkage is described as mostly influenced by basic density whereas in longitudinal shrinkage fibre organization is known to prevail. At the microscopic scale, the wood cell wall is first made of a middle lamella, which acts as joint between cells, then a thin primary wall and a thick secondary wall. Secondary wood cell walls are divided into three layers, an outer S1 with transversely oriented cellulose microfibrils, a thick S2 layer with microfibrils oriented at an angle (MFA) varying from 10 to 30° from the cell axis, and an inner S3 layer also with more transversely oriented microfibrils [6, 7]. Cellulose microfibrils are embedded in a matrix of lignin and amorphous polysaccharides [8] characterized by swelling potential. S1 and S3 layers are relatively thin, but play an important role in strengthening the cell against transversal deformation such as during drying shrinkage under varying humidity conditions [9]. Nonetheless, the behaviour of the S2 layer, being much thicker than the others, dominates the physical properties of the cell wall [10, 11]. Yamamoto et al [11] show that, with basic density, microfibril angle in the S2 layer is strongly related to the longitudinal modulus of elasticity and to longitudinal drying shrinkage: low MFA influences tangential shrinkage, while high MFA (30–40°) influences longitudinal shrinkage.

Secondary metabolites in heartwood are mostly formed in the transition zone during heartwood formation (*Robinia*-Type) [12]. The biosynthesis of extractives in sapwood (*Juglans*-Type) can also occur, but this has only been identified in a limited number of species [13]. Newly formed extractives diffuse from their production site (parenchyma cells) to the lumen before crossing cell membranes [14]. Metabolites partly fill the mesopores of the heartwood cell walls during the transformation from the sapwood [15] and are quantitatively and qualitatively quite different among species. These small molecules have different functions, contributing to plant fitness and impacting variability of wood properties of interest for industrial and commercial purposes. The effects of chemical extraction on drying shrinkage have been investigated for several species [16 –19] demonstrating that the removal of wood extractives impacts shrinkage behaviour. Presumably sorption sites formed after metabolite extraction represent additional vacant spaces responsible for tangential dimension increase [20, 21]. In 1986, the study of twelve *Eucalyptus* species after chemical extraction [5] revealed positive and negative correlations between extractive content and shrinkage, dependent upon which solvent was chosen. This indicates that all metabolites do not have the same effect. Therefore the need to identify biomarkers of dimensional stability is now of interest. Usually the effect of extractives on wood shrinkage is studied by comparing dimensional variability during the drying process of twin samples, one raw and one extracted. However removing extractives damages the wood structure, impacts the shrinkage behaviour and disrupts cell wall behaviour [22]. Dimensional variability of extracted samples may therefore not be fully representative of the influence of extractive compounds on wood shrinkage.

The objective herein is to study the influence of basic density and extractive content, as well as interlocked grain and MFA, on the drying shrinkage of *B*. *guianensis*, described as a “paradoxical” species given its singular behaviour. Contrary to most fast growing species, generally associated with low wood basic density and low durability, *B*. *guianensis* is a fast growing heliophilous species with excellent middle basic density and high durability. Its dimensional stability is an unexpected technical characteristic for fast growing species. Previously, heartwood extractives of *B*. *guianensis* have been studied for their contribution to wood durability [23] but their impact on shrinkage is unknown so far. In this study we measure shrinkage, basic density and extractive content in order to gain insight into the dimensional stability of *B*. *guianensis*.

## Materials and Methods

### 1. Samples collection

*Bagassa guianensis* Aubl. trees were sampled in a natural secondary forest near the Paracou experimental station (5°16'27N; 52°55'26W) [24], in French Guiana, in a field that belongs to CIRAD institution which is dedicated to experimental researches. *B*. *guianensis* (commercially known as Tatajuba), grows in secondary and or anthropized forest. All individuals (N = 11) have homogeneous size distribution in diameter from 13 cm to 55 cm. Samples were collected at three different heights (1.30 m, mid-height, under the crown) of the trunk and diametric planks were carefully sampled in order to keep constant orientation for each height. Longitudinal, radial and tangential shrinkage were measured along the diameter of each plank from bark to bark every 2.5 cm. Experiments allowing the comparison of drying shrinkage, basic density and extractive content were performed on wood from a board sampled at 1.3 m height in an additional individual of 22 cm of diameter. This tree was chosen with a regular shape, in order to reduce the probability of growth defects and singularities. We used a board at 1.3 m height and carried out four close matched samples. Chemical extraction and physical properties were measured on twin samples (on the same growth ring), as detailed in the sampling scheme (S1 Fig).

The originality of this method is the use of twin samples for each measurement, which allows preserving the integrity of the structure of each sample.

### 2. Shrinkage measurement protocol

Dimensional variations during the drying process were calculated for each orthotropic direction. The methods used to conduct this analysis were selected to fit the different scales of observation. Longitudinal shrinkage measurements were adapted from Clair et al [25] and performed on large samples (RxTxL = 20x20x80mm) using pinpoint measuring devices fixed to a metallic frame in which samples were secured. Radial and tangential shrinkage measurements were performed on small samples (RxTxL = 20x20x10mm). The total volumetric shrinkage used to characterize each sample is computed as the sum of longitudinal, radial and tangential strains. Saturated conditions were ensured by suspending samples in water after sawing. Samples then passed through four different moisture content (MC) stages: saturated, 10% (+/-1%) MC in a climate controlled room, 6% (+/1%) MC in an enclosure with MgCl_2_ salts solution, and in an oven-dried state reached after 3 days at 103°C.

### 3. Basic density measurement protocol

Basic density (BD) is defined as the ratio between dry mass (M0%) and saturated volume (V_Sat_) Eq (1) [26, 27]. Measurements were performed on small samples (RxTxL = 20x20x10 mm). Sample volume (V_Sat_) was calculated using an inversed Archimedes Principle method on a Sartorius CP224S balance (precision: 0.2 mg). For each step of stabilization, the real MC was calculated at the end, after measurement of M0% Eq (2). In order to take into account the influence of extractive content on wood basic density, we calculated a corrected basic density (excluding extraneous substances (BDc) to be used instead of BD as recommended by Hernandez [28] Eq (3). This corrected basic density is independent of the extractive content of the material and can be seen as substantive structural information.

$$BD=\frac{M0\%}{V_{Sat}}=\;\frac{M0\%}{Mv-Mi}$$ (1)

$$MC=\frac{Mx-M0\%}{M0\%}$$ (2)

$$BDc=\frac{M0c}{V_{Sat}}=\frac{M0\text{\%}}{V_{Sat}}\left(1-EC\right)=SG*\left(1-EC\right)$$ (3)

With BD = Basic density, MC = moisture content, BDc = corrected Basic density, M0% = oven-dry mass, VSat = saturated volume, Mv = fresh mass, Mi = immersed mass, Mx = mass at given moisture content, M0c is the oven-dry mass without extractive content, EC = extractive content.

### 4. Fiber saturation point (FSP)

FSP is defined as the moisture content at which the cell wall is still saturated but no more free water remains in the lumen. Practically, it is considered as the moisture content at which wood samples start to shrink. It is determined as the y-intercept of the linear regression between tangential shrinkage and MC measured at 10% MC, 6% MC and oven-dry. As for BD, a corrected FSP (FSPc) was calculated by subtracting the mass of extractives from the dry mass. FSP is therefore function of cell wall MC (MC_CW_), whereas FSPc refers to the moisture content of the lignocellulosic material (MC_LCM_) within cell wall (excluding extractives).

### 5. Interlocked grain and MFA

Interlocked grain was measured by visual assessment with a graduated goniometer on samples used for density measurement. Microfibril angle (MFA) measurements were carried out using X-ray diffraction (XRD) following same procedure as described previously [29].

### 6. Chemical extractions, HPLC and NMR profiling

Samples were stabilized at MC 10% (± 1%), in a climate controlled room and ground into a coarse powder. Next, a centrifugal mill (0.2 mm) was used to produce a homogeneous fine powder, which optimizes extraction efficiency and process repeatability. Powders were dried at 60°C for two days and two replicates per sample were performed. Each sample of dry wood powder (2 g) was introduced into a 150 ml Erlenmeyer and then 50 ml of water/methanol solution (ratio 1:4) was added. The wood material was dynamically macerated on a mechanical stir plate for 48h at room temperature (25°C). After filtration on a sintered-glass filter, the organic phase was concentrated under low pressure with a rotatory evaporator (Heidolph Laborota 4000) below 37°C. The concentrated extract was transferred into a test tube and evaporated at 40°C using a speedvac concentrator (Savant SPD121P, Thermo Scientific). Finally samples were dried under high vacuum (10^−3^ bar) for 2 hours. Test tubes were weighed on a precision balance to calculate the final extractive content (EC).

To perform Nuclear Magnetic Resonance (NMR) analysis, 50 mg of extract was dissolved in deuterated methanol (CD_3_OD) and filtered (0.22 μm) prior to introduction into the NMR test tube. Spectra (^1^H, ^13^C, HMBC) were acquired on a Varian spectrometer operating at 399.834 MHz proton frequency at 25°C. Water suppression was achieved by applying presaturation. For proton ^1^H NMR spectroscopy 64 transients were recorded with an acquisition time of 2 s and a relaxation delay of 2.0 s. For HMBC spectra, 64 × 256 increments were recorded in a spectral width of 6410.3 Hz with a relaxation delay of 5 s. All NMR spectra were phased and baseline corrected with MestReNova software (version 6.0.2; Mestrelab Research SL, Santiago de Compostela, Spain).

HPLC samples were prepared from extracts diluted in methanol (10 mg/mL), filtrated (0.22 μm) before injection (5 μL) in Varian 920-LC system equipped with UV-VIS detector and a photodiode array detector (C_18_ Discovery column, Sigma, 5 μm, 4.6 ×150 mm, flow rate 1 mL/min). All samples were analysed using a linear gradient of H_2_O/CH_3_CN/formic Acid (95:5:0.1 to 5:95:0.1).

### 7. Statistical analysis

We developed simple and multiple linear models using *lm* function in R statistical software [30]. For all fitted model, variance homoskedasticity and residuals normality were checked. The relative importance of each predictor per model was computed with the *relaimpo* function available in R package “relaimpo” [31].

## Results

### 1. Basic density and shrinkage

Basic density of *B*. *guianensis* revealed a significant and rarely observed wood variability within the trunk. Indeed we recorded extreme values from 0.256 g.cm^-3^ near the pith to 0.894 g.cm^-3^ in samples close to the bark. No significant variation was detected (ANOVA, df = 1, F = 1.04, p.v. = 0.31) between the 11 trees sampled. On the contrary, we recorded a similar density profile for each trunk. Based on 726 wood samples from 11 trees, we obtained a mean value of 0.59 g.cm^-3^ (median: 0.61 g.cm^-3^) with standard error of 0.007. Variations within tree heights were negligible compared to the radial gradient.

The mean volumetric shrinkage is 8.67% (SD = 2.4) for the entire sample set. Surprisingly, while radial density displayed a high gradient, we measured homogeneous shrinkage. Significant difference was observed between heartwood and sapwood samples where heartwood samples have low deformations (volumetric shrinkage of 7.28%) whereas sapwood samples are characterized by higher values (volumetric shrinkage of 11.87%) (Table 1). Shrinkage anisotropy was found to be very low with a mean ratio of 1.37 and a mean difference of 1.49 between tangential and radial shrinkage. Ratio only slightly differs from sapwood to heartwood whereas the difference T-R appears significantly lower in heartwood. Such low difference between T and R shrinkage indicates that this wood will be less prone to distortion and cracks during drying.

**Table 1:** Radial shrinkage, tangential shrinkage, ratio tangential/radial (T/R), difference tangential-radial (T-R), longitudinal shrinkage and total volumetric shrinkage of *B*. *guianensis* wood at three heights (1.30 m, mid-height, under the crown). Mean values for 11 individuals. SD are presented in parentheses.

*Shrinkage measures*	Radial (%)	Tangential (%)	T/R	T-R	Longitudinal (%)	TOTAL (%)
All samples	**3.60** (*1*.*3*)	**4.94** (*1*.*3*)	1.37	**1.49**	**0.24** (*0*.*2*)	**8.67** (*2*.*4*)
Sapwood	**4.96** (*1*.*1*)	**6.61** (*0*.*7*)	1.33	**1.69**	**0.31** (*0*.*3*)	**11.87** (*1*.*7*)
Heartwood	**2.93** (*0*.*7*)	**4.13** (*0*.*6*)	1.41	**1.35**	**0.19** (*0*.*2*)	**7.28** (*1*.*6*)

**Fig 1:**
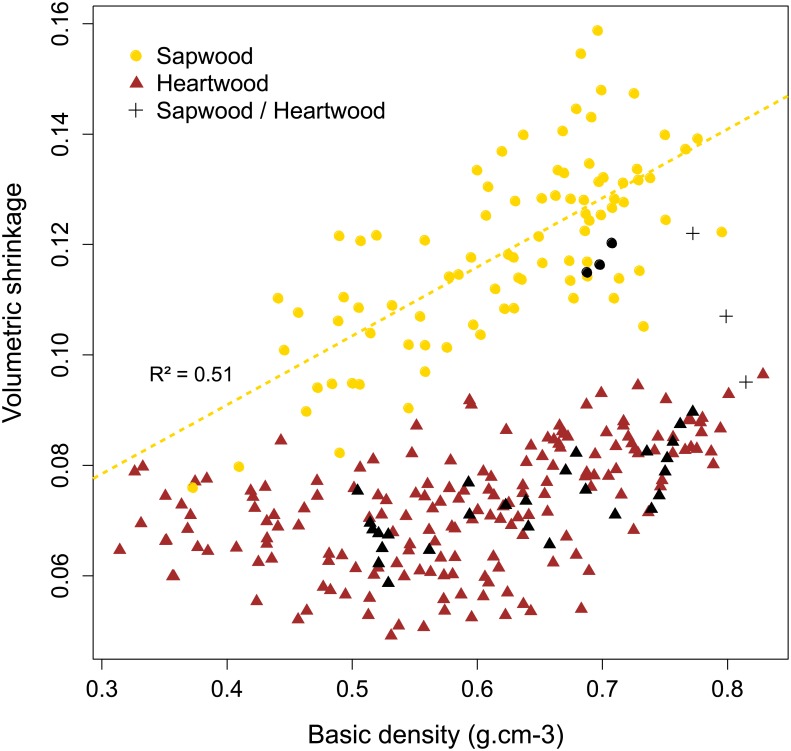
Influence of basic density on total volumetric shrinkage in sapwood and heartwood samples for 11 individuals of *B*.*guianensis*. Black symbol represent samples used for extraction experiments.

The influence of BD on total shrinkage differs considerably between sapwood and heartwood. Fig 1 illustrates the influence of BD on total shrinkage both in sapwood and heartwood. In sapwood, subsampling of BD and shrinkage are linearly correlated (intercept = 0,041; coefficient = 0.125; p-value = 8.66e-15; R^2^ = 0.51), whereas in heartwood samples no clear linear trend could be pointed out.

**Fig 2:**
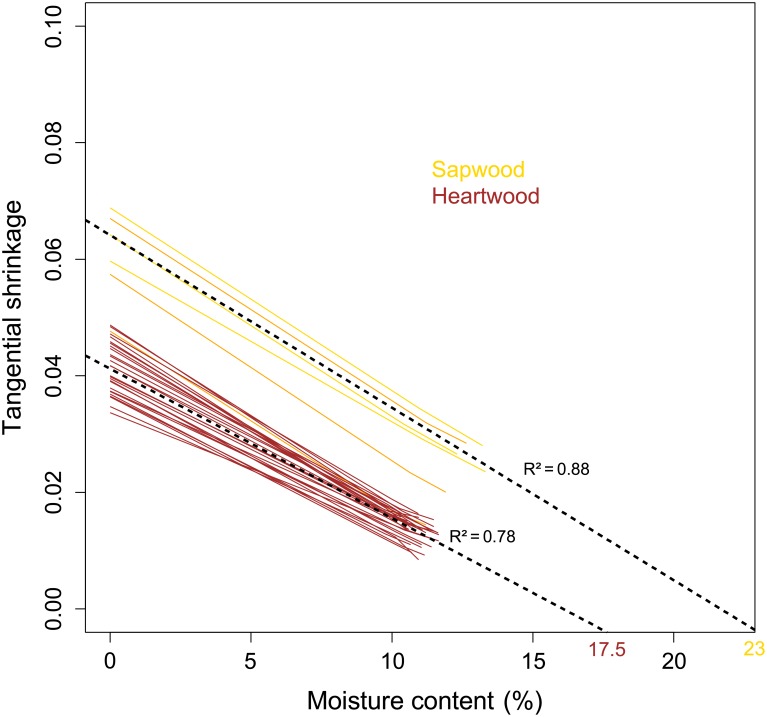
Relationship between tangential shrinkage and moisture content (MC) for sapwood and heartwood subsampling of *B*. *guianensis* used for chemical extractions (N = 33). Yellow and brown lines respectively correspond to sapwood and heartwood measures and black dashed lines indicate the mean values calculated for each subsampling.

FSP values also evidences clusters between wood types (Fig 2). FSP is significantly higher in sapwood (mean value: 21%) than in heartwood (mean value: 17%) and within the subsampling used for chemical extractions, mean FSP value was 23% in sapwood and 17.5% in heartwood. From this subsampling where extractive contents are known, FSPc values are 20% in HW and 23.5% in SW.

### 2. Interlocked grain and MFA

*B*. *guianensis* is characterized by a high variability in grain orientation within the radial profile. The interlocked grain angle is initially low near the pith and regularly increases towards the bark. In larger specimens, the interlocked grain angle can reach 25°. Profiles observed for the 11 individuals were very similar despite the strong differences in stages of development or growing conditions between trees. Contrary to interlocked grain, the X-ray measurements revealed that MFA was mainly constant in mature wood with little variation around the mean value of 11.6° (variance of 3.6° obtained with 548 samples measured). MFA was slightly higher (20°) in the first 5 cm around the pith, with similar results for juvenile wood.

Despites this high variability, we did not notice any significant effect of the grain angle on shrinkage for the whole dataset or for sapwood and heartwood subsamples. A linear model using grain angle as a factor was not valid to explain either radial or tangential shrinkage (respectively: R² = 0.001, p-value = 0.68; R² = 0.002, p-value = 0.61). Similarly, statistical analysis did not reveal any correlation between MFA and shrinkage in each three directions.

### 3. Phytochemical analysis

**Fig 3:**
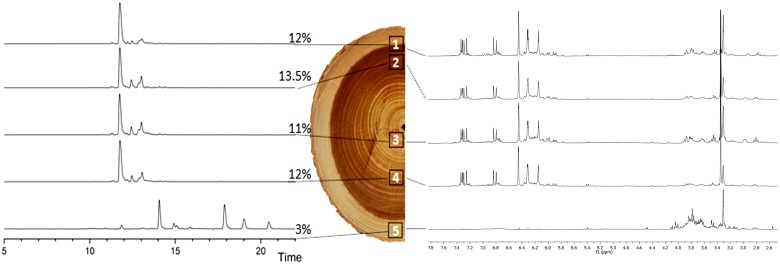
Stacked view of HPLC chromatograms (A), ^1^H NMR spectra of heartwood and sapwood extractives (C) and corresponding extraction yield (B).

Extractive content of heartwood samples (N = 9) from the *B*. *guianensis* individuals used for extraction analysis were qualitatively and quantitatively similar, with a mean value of 12.17% (Fig 3). Proton (^1^H) and proton-carbon correlation (^1^H-^13^C HMBC) NMR spectra revealed signals corresponding to aromatic compounds, one glycoside compound, fatty acids, and/or esters (S1 and S2 Figs). Additionally, ^13^C signals from olefinic double bonds were correlated with fatty derivatives proton signal and several correlations reflect the presence of glycosylated phenylpropanoids. HPLC chromatograms confirmed the presence of multiple molecules. Based on previous phytochemical studies [32] *trans*-oxyresveratrol was identified as the major phenylpropanoid product of heartwood extractives. In contrast sapwood sample 5 in Fig 3 contained only 2.75% extractive content, and its composition differs. ^1^H and HMBC NMR spectra show the presence of carbohydrates as major metabolites along with a small amount of phenylpropanoids, including *trans*-oxyresveratrol, and saturated fatty acids (S3 and S4 Figs). We did not observe olefinic double bonds of fatty derivatives previously identified in heartwood EC. Sapwood HPLC chromatogram revealed the presence of more lipophilic molecules and the absence of heartwood metabolites. Sample 1, a mix between sapwood and heartwood, gives similar results to exclusively heartwood samples i.e. high extractive content (11.78%) that are mostly composed of *trans*-oxyresveratrol.

All extractive content measurements from heartwood and sapwood are supported by high levels of reliability between two replicates (max diff. = 1.05% on average) and low rates of experimental incertitude (0.38%).

**Fig 4:**
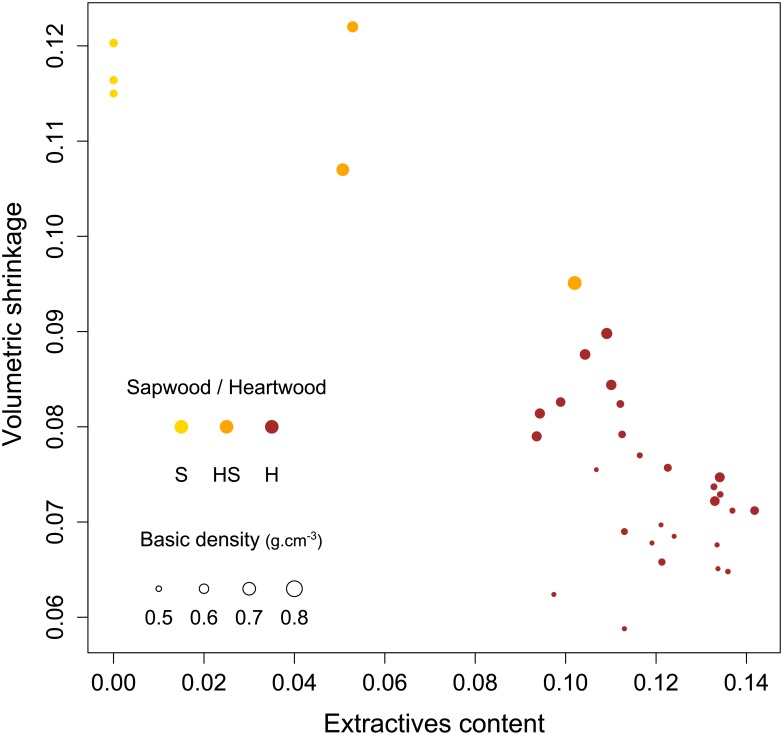
Effect of extractive content on the total volumetric shrinkage for one individual of *B*. *guianensis*. Basic density is represented by the thickness of the circles and colours correspond to different wood types.

Fig 4 shows the volumetric shrinkage of one individual as a function of extractive content and basic density. The shrinkage decreases from 12% to 6%, which correlates with extractive content increasing. The highest shrinkage value corresponds to sapwood sample 1 with the lowest extractive content.

### 4. Statistical models

**Fig 5:**
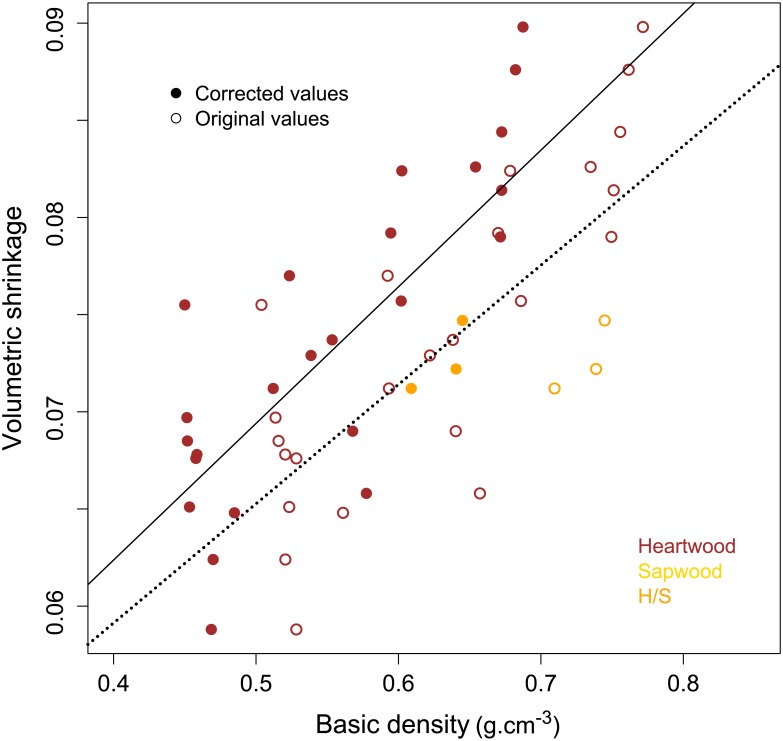
Relationship between volumetric shrinkage and basic density (empty circles) or corrected basic density (filled circles) in *B*. *guianensis*.

The variance in the simple linear model using BD or extractives separately explained, respectively 35.9% and 44.9% of the shrinkage variability. In order to better account for the original heartwood structure, the density can be corrected to eliminate the additional mass of secondary metabolites. Fig 5 compares the linear models obtained using BD and corrected BD (BDc) in correlation to volumetric shrinkage. The shrinkage predictive model is significantly improved (r² = 0.38 without correction; r² = 0.53 using BDc).

To better understand which parameter is involved in the volumetric shrinkage, three different multilinear models were tested. Models 1 (m1) and 2 (m2) used extractive content and, respectively, BD or BDc as independent variables. The third model (m3) differentiated three variables: BDc, sapwood extractive content (ECs) and heartwood extractive content (ECh). The final model (m3_h) was restricted to heartwood subsampling and used BDc. Statistical results are summarized in Table 2.

**Table 2:** Multiple linear models linking shrinkage with basic density (BD) and extractive content (EC). Intercepts, estimate (Est.), standard errors (SE), t and p values and significance levels (Sig: **:0.01–0.001; ***<0.001) are given for each predictive variable. Sample size (N), adjusted explained variance (R2) and degrees of freedom (df) are given for each model.

models	m1					m2					m3					m3_h				
	n	df	R²			n	df	R²			n	df	R²			n	df	R²		
	33	30	0.71			33	30	0.71			33	29	0.91			30	24	0.65		
	Est.	SE	t	p	Sig	Est.	SE	t	p	Sig	Est.	SE	t	p	Sig	Est.	SE	t	p	Sig
**Intercept**	0.06	0.01	4.25	0	***	0.05	0.02	3.34	0	**	0.07	0.01	7.52	0	***	0.05	0.01	4.15	0	***
**BD**	0.09	0.02	5.02	0	***	-	-	-	-	-	-	-	-	-	-	-	-	-	-	-
**BDc**	-	-	-	-	-	0.1	0.02	5.04	0	***	0.06	0.01	4.68	0		0.06	0.01	5.72	0	***
**EC**	0.34	0.06	-5.85	0	***	-0.27	0.06	-4.38	0		-	-	-	-	-	-	-	-	-	-
**EC_s**	-	-	-	-	-	-	-	-	-	-	-0.22	0.03	-6.17	0	***	-	-	-	-	-
**EC_h**	-	-	-	-	-	-	-	-	-	-	0.31	0.08	3.89	0	***	-0.13	0.08	-1.67	0.1	

All three models predict the drying shrinkage with at least 65% of the variance explained. The use of BDc instead of simple BD (m1 and m2) results in a low gain of 0.1 point. However m3 model, which differentiates heartwood and sapwood extracts, increases notably by more than 20 points to reach 91% of variance.

**Fig 6:**
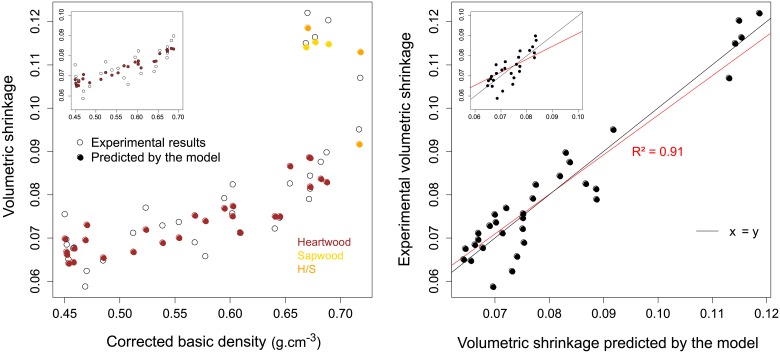
Relationship between total volumetric shrinkage and corrected specific density in sapwood, heartwood and mixed samples. Empty circles and full circles represent experimental results and predictions from the model m3 respectively. On the top-left corner is presented the heartwood-subsampling predictions using model m3_h.

When heartwood samples are analysed independently, extractive content appears as an explanatory factor. Does the presence of extractives minimize shrinkage in a binary manner or is shrinkage quantitatively linked to the extractive content? The model (m3_h) is less explicative (65%) than m3, however, it confirms that drying shrinkage in heartwood is highly correlated to extractive content. To illustrate these results, comparison between predictions and observations are depicted in Fig 6.

## Discussion

### 1. Heartwood and sapwood: two different shrinkage behaviours

Analysis of 11 tree individuals shows that heartwood commonly represents 70% of the radial profile at breast height. Heartwood is therefore subject to a large radial gradient impacting its properties. The density variability is higher in heartwood than in sapwood, but shrinkage nonetheless remains homogeneous all along the heartwood samples. Indeed, internal and external heartwood deformations were equivalent between samples despite having strong variation in density from juvenile wood to mature wood. Also, samples with similar BD value showed contrasted results depending on wood type. For example, heartwood and sapwood samples with the same BD of 0.6 g.cm^-3^ have a volumetric shrinkage ranging from 5.2% to 9% and 10.5% to 13.8% respectively, suggesting that low extractive content in sapwood do not prevent shrinkage.

**Fig 7:**
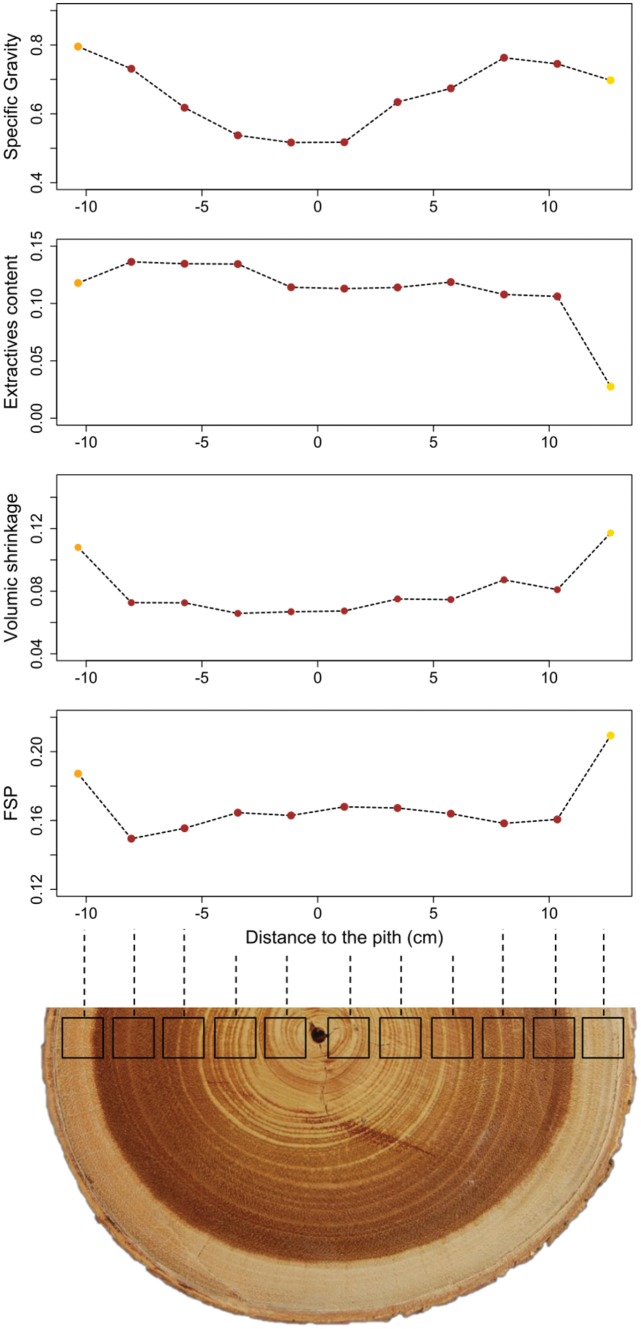
Stacked view of basic density, extractive content, shrinkage and FSP along one individual truck cross-section.

To illustrate the remarkably low and homogeneous shrinkage of heartwood samples, a stacked view of BD, shrinkage, FSP and EC for the individual used for chemical extractions is presented in Fig 7. Clearly the shrinkage profile is driven principally by extractive content. Indeed in the heartwood, the shrinkage is homogeneous as well as extractive content whereas BD varies from 0.52 g.cm^-3^ near the pith to 0.79 g.cm^-3^ in samples close to sapwood. Secondly, one can also observe the dependence of shrinkage upon basic density within the heartwood samples. FSP results also evidenced clear heartwood/sapwood dissociation, with different responses to the drying process.

We did not observe any significant correlation between interlocked grain and shrinkage nor between MFA and shrinkage in each 3 directions (data not shown). This is in accordance with Yamamoto’s work [11], that evidenced the contribution of MFA to shrinkage only for MFA values under 5° (transversely) or over 30° (longitudinally).

### 2. Coupled effect density—extractives

Physical and chemical analyses were conducted in parallel on twin samples. This approach allowed us to reconsider BD measurements. Extractives played a direct role in the dimensional stability of wood samples during the drying process. It was necessary to take into account the amount of extractives in samples in the model as a consistent parameter to explain more precisely the variability of this species regarding shrinkage behaviour. A mixed model using both BDc and EC is the best adapted to predict shrinkage for *B*. *guianensis*. In the final model m3, BDc explained 23.5% of the variance whereas extractives explained 67.8%. Within heartwood, where EC variability between samples was low compared to changes in density, the rate of variance of explanation from the chemical characteristics is reduced and BDc becomes the most impacting variable.

### 3. Localization of the biosynthesis machinery in *B*. *guianensis* wood

Heartwood secondary metabolites are crucial components that ensure the natural durability and the mechanical stability of heartwood. Better understanding the heartwood metabolites biosynthesis is crucial to lift the veil on the tree chemical strategy associated with the heartwood formation process. In *B*. *guianensis* sapwood and heartwood extractives are composed of the same class of metabolites (phenylpropanoids, carbohydrates, olefinic compounds), but in different ratios. Sapwood HPLC chromatograms reveal the absence of most heartwood metabolites, beside *trans*-oxyresveratrol, and the presence of more lipophilic molecules not detected in heartwood. We assume these lipophilic compounds could undergo enzymatic biotransformation cascade during the duraminisation process in addition to *de novo* synthesis in parenchyma cells, thus leading to the new formation of heartwood extractives. So far the detection of newly formed olefinic carbon-carbon double bonds was observed using the HMBC NMR experiment, and the main heartwood constituent *trans*-oxyresveratrol was detected in sapwood extractives. This implies that *B*. *guianensis* can be considered as a *Juglans*-Type heartwood formation.

### 4. Toward a comprehensive explanation of the drying process of *B*. *guianensis* heartwood

Considering our results, a new hypothetical mechanism of drying shrinkage in wood cell walls in *B*. *guianensis* can be proposed. This model must account for a given density for both: (i) a lower total shrinkage when amount of extractives increases; (ii) a lower FSP in heartwood compared to sapwood.

**Fig 8:**
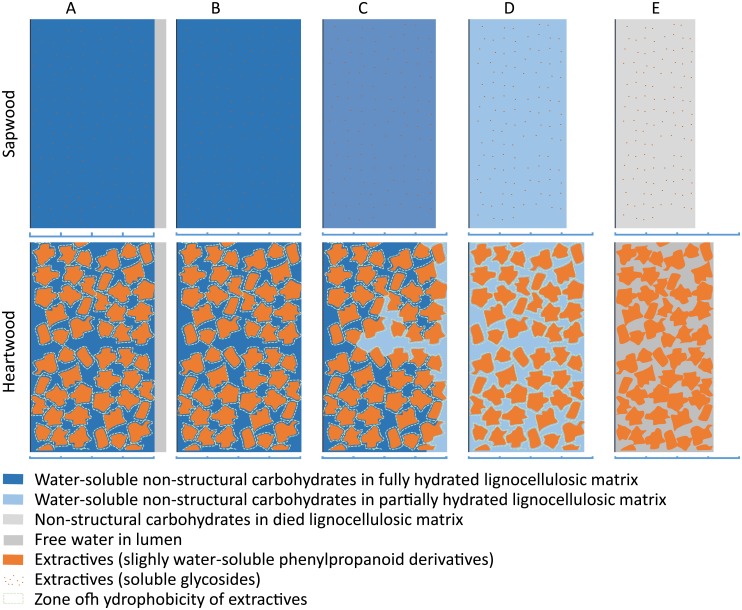
Tentative representation of the drying process of *B*. *guianensis*’s wood. A. Over FSP, cell wall fully hydrated, free water in lumen. B. Fibre saturation point (FSP), cell wall (CW) and lignocellulosic material (LCM) fully hydrated (MC_CW_~23%, MC_LCM_~23.5%), no water in the lumen. C. Bellow FSP (23% > MC_CW_ > 17.5%, 23.5% > MC_LCM_ > 20%), CW and LCM partially hydrated, sapwood (SW) shrinks, heartwood (HW) still un-shrunk. D. drying continuation (MC_CW_ < 17%, MC_LCM_ < 20%), both SW and HW shrink. E. Oven-dry, HW shrinkage prevented by steric hindrance of extractives. Levels of moisture contents used in this representation are proposed in accordance with measurement made on the sub-sampling used for extraction experiments.

The lower FSP in heartwood (17.5%) compared to sapwood (21%) supports the idea that extractives, during the maturation process, take the place of water sorption sites. Therefore, wood cell walls, even fully hydrated, contain less water in heartwood than sapwood. However, the calculation of FSPc, which takes into account the mass of extractives and therefore refers exclusively to the water content of the lignocellulosic material, shows that not only the cell walls but also the lignocellulosic material itself contains less water in heartwood than sapwood when the shrinkage starts (FSPc). From this first observation we can conclude that in heartwood, some water started to desorb without producing strains on the sample. This implies that the way to measure FSP, based on dimensional changes, would not be adapted to determine FSP in heartwood. We hypothesis that the "real" FSP (the moisture content at which the sample contains no more free water and the cell walls are still full saturated, 23% in our subsampling) would be the same in both sapwood and heartwood, but because of the presence of extractives, heartwood may lose bonded water without starting to shrink (Fig 8). We propose that a zone of hydrophobicity acts as a barrier in the vicinity of the extractives, preventing water diffusion in some parts of the cell walls when the water content is high. This assumption is supported by the lipophilic character of phenylpropanoid and fatty derivatives (>80% of heartwood extractives composition) that move as lipophilic droplets from their production site to heartwood tissues [33]. Metabolite diffusion into hydrated cell walls creates hydrophobic interaction with water thus leading to a heterogeneous mixture. Only water in the vicinity of the lumen, and when not trapped by groups of extractives, starts to dry. This small amount of water desorbed reduces the moisture content of the sample without changing cell wall dimensions.

When the sample continues to dry, the potential becomes high enough to let water pass through the hydrophobic barrier and the sample starts to shrink. Finally, lignocellulosic material continues to desorb but cell wall strains are blocked by the steric hindrance of extractives in the wall, resulting in a lower shrinkage in heartwood than sapwood; with the higher extractive content, the total shrinkage is lower.

## Conclusions

This study provides an unbiased method to evaluate how secondary metabolites impact wood properties. We demonstrate that *B*. *guianensis* present an excellent dimensional stability during heartwood drying process that could not be only correlated to basic density. Quantitative analysis of heartwood chemical extracts shows high extractive content that are clearly correlated to the low drying shrinkage. These results confirm that secondary metabolites must be taken in account in the characterisation of wood properties and future predictive models must integrate qualitative and quantitative analysis of their EC. NMR spectroscopy analysis demonstrates that heartwood metabolites biosynthesis begins in sapwood and most of production sites are located in the transition zone.

Finally the combination of rapid growth, high durability, low drying shrinkage and low anisotropy in heartwood makes *B*. *guianensis* a suitable species for industrial use. Moreover *B*. *guianensis* is adapted to growth in anthropized forest clearings and secondary forests, a clear advantage for future plantation projects. This Amazonian species deserves to be further studied and employed in tropical areas.

## Supporting Information

S1 Fig Sampling scheme used for the experiments allowing the comparison of drying shrinkage, basic density and extractive content. (EPS)

S2 Fig Proton NMR spectra of *B*. *guianensis* heartwood. (EPS)

S3 Fig HMBC NMR spectra of *B*. *guianensis* heartwood. (EPS)

S4 Fig Proton NMR spectra of *B*. *guianensis* sapwood. (EPS)

S5 Fig HMBC NMR spectra of *B*. *guianensis* sapwood. (EPS)
